# Low-Fat Cheddar Cheese Influences Gut Microbiota Composition and Diversity in Human Microbiota–Associated Mice

**DOI:** 10.3390/foods15010066

**Published:** 2025-12-25

**Authors:** Si Lu, Mairui Gao, Deepa Kuttappan, Mary Anne Amalaradjou

**Affiliations:** Department of Animal Science, University of Connecticut, Storrs, CT 06269, USA

**Keywords:** low-fat cheddar cheese, gut microbiota, human microbiota–associated mice, functional foods, probiotics, lactic acid bacteria, *Firmicutes*, *Bacteroidetes*, microbial diversity, 16S rRNA sequencing

## Abstract

Cheese is a complex fermented dairy food containing bioactive nutrients and microorganisms that can influence host physiology. However, most existing evidence of its health effects derives from observational studies or investigations of isolated components rather than the whole food matrix. The present study examined the impact of low-fat Cheddar cheese as a whole food on the gut microbiota using a human microbiota–associated (HMA) mouse model. Germ-free C57BL/6 mice were colonized with human fecal microbiota and randomly assigned to either a control diet or a diet supplemented with low-fat Cheddar cheese (7.5% *w*/*w*) for six weeks. Fecal samples were collected longitudinally and analyzed by 16S rRNA gene (V3–V4 region) amplicon sequencing. Human microbiota transplantation successfully established a stable, human-like gut microbial community in the mice. Cheese supplementation significantly increased alpha diversity (Shannon and Chao1 indices) and altered microbial composition, characterized by a higher relative abundance of *Firmicutes* and a reduction in *Bacteroidetes* (*p* < 0.001). At the genus level, *Lactococcus* and *Streptococcus* were enriched in cheese-fed mice, reflecting potential viable transfer of cheese-derived lactic acid bacteria. These findings provide experimental evidence that low-fat Cheddar cheese can beneficially influence the human-derived gut microbiota in an animal model and highlight the need for further clinical research to validate these effects in humans.

## Simple Summary

Cheese is one of the most widely consumed fermented dairy foods, yet there is limited experimental evidence on how it influences the gut microbiota. Most previous studies have relied on population surveys or on testing isolated cheese components, rather than cheese as a whole food. In this study, we investigated how daily consumption of low-fat Cheddar cheese affects the gut microbiota using a specialized mouse model colonized with human intestinal bacteria. Human microbiota–associated mice were fed either a standard diet or a diet containing low-fat Cheddar cheese for six weeks. We found that cheese consumption significantly changed the structure and diversity of the gut microbial community. The cheese-fed mice showed an increase in overall bacterial diversity and a greater abundance of *Lactococcus* and *Streptococcus* microorganisms naturally present in cheese and known for their potential probiotic properties. These results suggest that cheese, when included as part of a balanced diet, can beneficially influence the gut microbiota. However, because this work was conducted in an animal model, further clinical studies are required to confirm whether similar effects occur in humans.

## 1. Introduction

The gut microbiota, defined as the collection of microorganisms and their interacting genomes within the gastrointestinal tract (GIT), plays a central role in host health [1,2,3]. The human GIT is colonized by approximately 100 trillion microorganisms, of which bacteria are the predominant members [4,5]. The maintenance of a stable microbial balance, primarily governed by commensal microbiota, is essential for gut homeostasis—characterized by resilience and resistance to external and endogenous disturbances [6]. In mammals, the gut microbiota is mainly composed of the phyla *Firmicutes*, *Bacteroidetes*, *Actinobacteria*, and *Proteobacteria* [7]. The most abundant bacterial families in the GIT include *Lachnospiraceae*, *Ruminococcaceae*, *Prevotellaceae*, *Bacteroidaceae*, and *Rikenellaceae*, although the detailed composition can vary among individuals [8,9]. This complex microbial community supports host health by forming a protective barrier against invading pathogens, regulating immune responses, and producing bioactive metabolites such as vitamins and short-chain fatty acids (SCFAs) [10,11]. Beyond the intestine, the gut microbiota influences extra-intestinal organs, including the liver, kidneys, and brain, thereby participating in systemic homeostasis [1,12]. Given its multifaceted role, alterations in the gut microbial composition (dysbiosis) have been associated with several pathological conditions, including obesity, metabolic syndrome, colorectal cancer, and inflammatory bowel disease [13,14,15,16,17].

Among the various factors influencing gut microbiota composition, diet represents the primary determinant [11,18,19,20]. Diet and health are inherently interdependent, and this relationship is particularly evident in the gastrointestinal tract, where most host–diet interactions occur [21]. Dietary modulation has been shown to prevent several chronic diseases, including allergy, obesity, inflammation, and cancer [22,23]. Many of these beneficial effects are increasingly attributed to the plasticity of the gut microbiota, which can rapidly adapt to dietary changes [24,25]. Consequently, the ability of the diet to modulate microbial composition and promote gut health is now recognized as a practical and promising prophylactic strategy to address multiple global health challenges.

Beyond their nutritional value, foods are increasingly recognized for their health-promoting properties, driving growing interest in the use of functional foods as prophylactic alternatives to pharmacotherapy [26,27]. Functional foods typically contain one or more bioactive components, including prebiotics, probiotics, antioxidants, polyphenols, bioactive amines, sterols, and carotenoids [26,28,29]. A key mechanism through which functional foods confer health benefits is by beneficially modulating the gut microbiota [24,25,30]. Additionally, these foods promote health by influencing cellular processes, nutrient metabolism, cell signaling, apoptosis, angiogenesis, and host metabolomics [21,26,31,32]. Among various dietary sources, dairy products, particularly cheese, have been identified as functional foods with diverse and well-documented health benefits.

Cheese is one of the most popular fermented dairy products worldwide, second only to yogurt, owing to its versatility and associated health benefits [33]. Among the various cheese types, cheddar cheese is particularly prominent, accounting for approximately 52% of total cheese consumption in the United Kingdom, while annual cheddar cheese consumption in the United States is estimated at around 1520 kt [34]. Nutritionally, cheese is an excellent source of high-quality protein, essential amino acids, bioactive peptides, conjugated linoleic acids, sphingolipids, minerals (including calcium, phosphorus, zinc, and magnesium), and vitamins (A, B_2_, B_6_, and B_12_) [35,36,37]. Beyond its nutritional composition, several studies have demonstrated the functional health benefits of cheese, including antimicrobial, antihypertensive, anti-inflammatory, antithrombotic, and anticancer properties [36,37,38,39,40,41,42].

In addition to its nutrient content, the cheese fermentation and ripening process endows it with a diverse microbial community, many members of which exhibit probiotic potential. These microbes contribute to gut health through mechanisms such as pathogen inhibition, colonization resistance, competitive exclusion, anti-inflammatory and antioxidant activity, and immunomodulation [43,44,45]. The beneficial effects of cheese-derived microorganisms are mediated via the secretion of antimicrobial peptides and short-chain fatty acids (SCFAs), reduction in luminal pH, enhancement of gut barrier integrity, and modulation of cytokine production and T cell distribution [46,47,48,49].

Given its functional properties, cheese supplementation has been investigated in several disease models, including ulcerative colitis, hyperlipidemia, and inflammatory bowel disease (IBD), for its potential health benefits [50,51,52,53,54]. In a clinical study, obese hypertensive patients consuming cheese containing *Lactobacillus plantarum* TENSIA exhibited an increased abundance of intestinal *Lactobacillus* spp., which was associated with a significant reduction in morning blood pressure [55]. Similarly, Veiga et al. [25] reported that consumption of a fermented milk product containing *Bifidobacterium animalis* enhanced short-chain fatty acid (SCFA) production and reduced the abundance of the pathobiont *Bilophila wadsworthia* in individuals with irritable bowel syndrome (IBS). In a murine model, Plé et al. [53] demonstrated that dietary supplementation with cheese containing *Propionibacterium freudenreichii* conferred protection against acute colitis through anti-inflammatory mechanisms.

Although most studies on functional foods have focused on disease models, comparatively few have examined their effects in healthy subjects. Lay et al. [56] were among the first to investigate the influence of Camembert cheese on the gut microbiota of human microbiota–associated (HMA) rats. Subsequently, Firmesse et al. [57,58] reported that regular consumption of Camembert cheese for two weeks significantly increased the abundance of commensal bacteria, including *Enterococcus faecalis*, *Lactococcus lactis*, and *Leuconostoc mesenteroides*, in healthy human volunteers. Likewise, Milani et al. [59] demonstrated that *Bifidobacterium* species present in Parmesan cheese transiently colonized the gut of healthy human subjects. Given the widespread dietary recommendation for the inclusion of dairy products in a balanced diet, along with the popularity and rich microbial diversity of cheese, its incorporation may beneficially modulate the gut microbiota. However, there remains limited research on the impact of low-fat Cheddar cheese on the gut microbiota in healthy models. Therefore, the present study aimed to investigate the effects of daily supplementation with low-fat Cheddar cheese, equivalent to two standard daily servings, on the gut microbiota of human microbiota–associated mice.

## 2. Materials and Methods

### 2.1. Human-Microbiota Associated (HMA) Mice Model

No human subjects research was conducted in this study. The investigators did not collect, handle, or process human materials at any time. Human fecal microbiota used for mouse colonization were obtained as fully anonymized, de-identified samples from a commercial vendor (OpenBiome, Cambridge, MA, USA), collected under the vendor’s IRB-approved protocols with informed consent. Colonization of germ-free mice was performed by Taconic Biosciences as a contracted service under their established regulatory oversight. All animal procedures conducted by the authors were reviewed and approved by the University of Connecticut Institutional Animal Care and Use Committee (IACUC). Female mice were exclusively used to minimize variability associated with sex-specific differences in diet-induced modulation of the gut microbiota. Sex hormones are known to influence both microbial composition and metabolic responses to dietary interventions, potentially confounding data interpretation when both sexes are included [60,61].

Twenty female C57BL/6 germ-free mice (3–4 weeks of age) were obtained from Taconic Biosciences (Rensselaer, NY, USA) and transferred to Taconic’s isolator breeding solutions facility. The mice were housed under germ-free conditions for 14 days and fed a double-irradiated AIN-95G control diet (Table 1). Humanization was achieved via fecal microbiota transplantation (FMT) using donor material obtained from OpenBiome (Cambridge, MA, USA). The OpenBiome sample originated from a healthy 24-year-old individual with a BMI of 24.3, no reported medications, no reported antibiotic or probiotic use, no known allergies, and a mixed diet. A single healthy human donor was used to minimize inter-individual variability in fecal microbiota composition, which can confound microbial transfer and interpretation of outcomes in HMA mouse models [62].

Colonization of germ-free mice was performed by Taconic Biosciences (Rensselaer, NY, USA) as a contracted service under their established regulatory oversight. Briefly, each mouse was inoculated via oral gavage with 100 µL of donor fecal suspension (1:5 dilution in sterile 1× phosphate-buffered saline) inside a biological safety cabinet. To promote microbiota establishment, a second inoculation was administered three days after the initial transplantation. A 14-day stabilization period was allowed to ensure successful colonization of the human microbiota, consistent with previous studies [62,63]. During this period, regular health monitoring, including body weight measurements and fecal sample collection, was performed.

**Figure 1:**
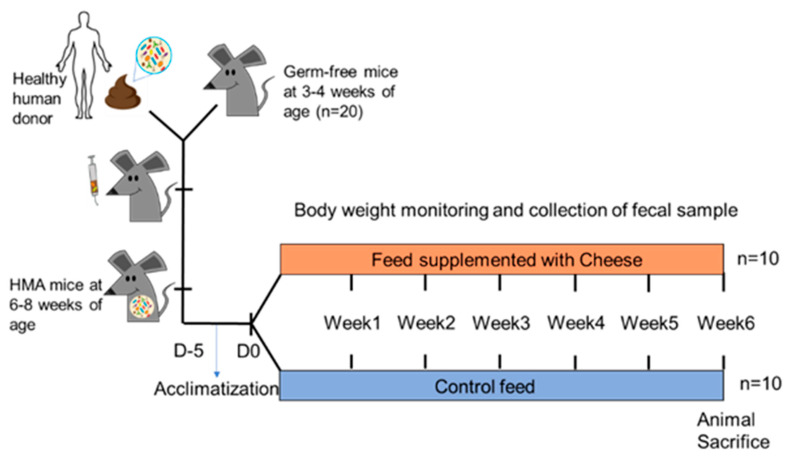
Schematic representation of the experimental design.

Following this 2-week period, animals were shipped in germ-free containers to the UConn vivarium. Upon arrival, mice were housed individually in sterile cages with ad libitum access to autoclaved water and the control diet. After a five-day acclimatization period, animals were randomly assigned to one of two groups: Control (*n* = 10) or Cheese (*n* = 10). Sample size (*n* = 10/group) was selected based on prior microbiome studies demonstrating that groups of 8–12 animals provide adequate statistical power to detect microbial differences at β = 0.8 and α = 0.05 [18,19]. Mice were maintained in the facility for the six-week feeding trial (Figure 1). Body weight was recorded at baseline and weekly thereafter. At the end of the trial, mice were euthanized by CO_2_ asphyxiation, and organ weights were measured [64].

Overview of the experimental setup to evaluate the effects of low-fat Cheddar cheese on gut microbiota in human microbiota–associated (HMA) mice. Germ-free C57BL/6 mice were colonized with human fecal microbiota and fed either a control diet or a diet containing 7.5% (*w*/*w*) low-fat Cheddar cheese for six weeks. Fecal samples were collected at baseline, week 1, and week 6 for microbiome analysis.

### 2.2. Cheese Manufacture

Low-fat Cheddar cheese was produced in a pilot-scale cheesemaking vat (Labtronix Inc., Monroe, OR, USA) using 5.91 gallons of pasteurized skim milk and 0.09 gallons of cream (Price Chopper, Schenectady, NY, USA) following the stirred-curd process. Calcium chloride (0.02% *w*/*w*; 34% solution) was added, and the milk was heated to 22 °C. The pH was adjusted to 6.2 using food-grade lactic acid (88%; Acros Organics, Waltham, MA, USA) diluted 1:1 with sterile deionized water. The milk was then warmed to 35 °C and inoculated with freeze-dried direct-vat-set lactic acid bacterial starter cultures *Lactococcus lactis* subsp. *lactis* (M58) and *Streptococcus thermophilus* (TA61) (Danisco A/S, Copenhagen, Denmark) at a rate of 4.25 units per 100 L of milk. After a 40-min ripening period, chymosin (DCI Star, Dairy Connection, Madison, WI, USA) diluted 1:10 with sterile deionized water was added at 8 mL/100 L, and the mixture was stirred for 45 s. The coagulum cutting time was determined using the formula: cutting time = flocculation time + (flocculation time × 1.5). Once the desired firmness was reached, the coagulum was cut into 1 cm^3^ curds. The curds were allowed to settle and heal for 5 min, then stirred for 40 min at 35 °C until a pH of 6.0 ± 0.05 was achieved, at which point whey drainage began. The curds were then stirred dry in alternating intervals (5 min on, 3 min off) until the pH reached 5.45 ± 0.05. Subsequently, the curds were washed twice with 12 °C water (500 mL each), thoroughly mixed after each wash, and drained. Salting was performed to achieve approximately 2.8–3 g of salt per kg of milk, resulting in a final cheese salt content of 1.8–1.9%. Salt was incorporated in three 10-min applications with continuous stirring. The curds were then transferred into rectangular molds (15 × 10.5 cm; M605MTF066, Fromagex, Rimouski, QC, Canada) and pressed overnight at room temperature under 60 lb of pressure (E28, New England Cheesemaking Supply, Northampton, MA, USA). The cheeses were removed from molds when a target pH of 5.2 ± 0.1 was reached. Finally, the cheese was grated, portioned into individual feeding pouches (3 mil, oxygen transmission rate: 50–70 cc/m^2^·24 h; UltraSource LLC, Kansas City, MO, USA), vacuum sealed (Ultravac 250, UltraSource LLC), and stored at 4 °C until use [65].

### 2.3. Experimental Diets

The basal diet for this study was based on the AIN-95G formulation (OpenSource Diets, New Brunswick, NJ, USA). This diet was modified to maintain isocaloric and iso-nutrient composition, as detailed in Table 1. The concentrations of major minerals, including sodium, potassium, calcium, magnesium, phosphorus, and iron, were adjusted by adding the corresponding raw materials to ensure compositional equivalence between the control and cheese-supplemented diets [66]. Low-fat Cheddar cheese was prepared using *Lactococcus lactis* subsp. *lactis* and *Streptococcus thermophilus* as starter cultures, following the stirred-curd process without a ripening stage, as described above. Pre-portioned grated cheese was finely pulverized using a food processor and incorporated into the basal diet at 7.5% (*w*/*w*) to formulate the cheese diet. The cheese-containing diet was prepared daily by mixing pre-portioned grated cheese into the basal mouse chow to preserve microbial viability and nutrient stability. The cheese inclusion level (7.5%) was determined based on the most recent U.S. Dietary Guidelines for Americans [67] and calculated using the body surface area (BSA) normalization method to convert human daily cheese intake into an equivalent dose for mice [68,69]. This mouse-equivalent dosage was used to ensure physiologically relevant translation of human cheese consumption to the animal model. Based on the U.S. Dietary Guidelines for Americans recommendations, a cheese inclusion level of 7.5% (*w*/*w*) corresponds to approximately two servings of cheese per day for humans.

Formulation of the experimental diets provided to human microbiota–associated mice. The control diet was based on AIN-95G, while the cheese diet included 7.5% (*w*/*w*) low-fat Cheddar cheese incorporated into the basal formulation to ensure isocaloric and iso-nutrient content between treatments.

Proximate composition of the experimental diets provided to human microbiota–associated mice. The control diet was based on AIN-95G, while the cheese diet included 7.5% (*w*/*w*) low-fat Cheddar cheese incorporated into the basal formulation to ensure isocaloric and iso-nutrient content between treatments.

### 2.4. Sampling, Genomic DNA Extraction, and 16S rRNA Gene Sequencing

Fecal pellets were collected from all animals (*n* = 10 per group) at baseline (prior to the start of the feeding trial), after one week, and at the end of six weeks. Samples were placed in RNase-/DNase-free microcentrifuge tubes and immediately frozen at −80 °C until analysis. In addition, fecal samples from germ-free and human microbiota–associated (HMA) mice were collected at the Taconic facility and shipped to the University of Connecticut for comparative analysis. DNA extraction and sequencing were performed at the University of Connecticut Microbial Analysis, Resources, and Services (MARS) facility. DNA was extracted using the MoBio PowerMag Soil 96-Well Kit (MoBio Laboratories, Carlsbad, CA, USA) according to the manufacturer’s protocol. DNA concentrations were determined using the Quant-iT PicoGreen dsDNA Assay Kit (Invitrogen, Thermo Fisher Scientific, Waltham, MA, USA). The V4 region of the bacterial 16S rRNA gene was amplified using primers 515F and 806R containing Illumina adapters and dual indices [70]. PCR amplification was carried out with an initial denaturation at 95 °C for 3.5 min, followed by 30 cycles of 95 °C for 30 s, 50 °C for 30 s, and 72 °C for 90 s, with a final extension at 72 °C for 10 min. PCR products were pooled, quantified, and visualized using the QIAxcel DNA Fast Analysis System (Qiagen, Hilden, Germany). The pooled amplicons were cleaned using Omega Bio-Tek Mag-Bind Beads (Omega Bio-Tek, Norcross, GA, USA) according to the manufacturer’s protocol. The final library pool was sequenced on an Illumina MiSeq platform using a v2 2 × 250 bp kit (Illumina Inc., San Diego, CA, USA). DNA extraction blanks and no-template PCR controls were included and processed identically to biological samples. These controls yielded substantially fewer reads (<100), and ASVs detected in these controls were removed prior to analysis.

### 2.5. Bioinformatic Analysis

Raw sequence reads were processed using the DADA2 pipeline (v1.20.0) in R (v4.1.0; [71]) for quality filtering, denoising, and generation of amplicon sequence variants (ASVs) [72,73]. Briefly, paired-end reads were truncated at 240 bp (forward) and 160 bp (reverse) to remove low-quality bases based on the per-base quality profiles, and primer sequences were removed using the *trimLeft* parameter. Reads with more than two expected errors (*maxEE* = 2) or containing ambiguous bases were discarded. The DADA2 core sample inference algorithm was then applied to denoise and merge the paired-end reads, and chimeric sequences were removed using the consensus method. Taxonomic assignment of ASVs was performed using the SILVA reference database (v138_train_set).

Downstream analyses and visualization were conducted in R using the *phyloseq* (v1.34.0) and *ggplot2* (v3.3.3) packages [71]. ASVs classified as Archaea, Eukaryota, chloroplasts, or mitochondria were removed prior to analysis. Rarefaction curves were generated from the amplicon sequence variant (ASV) abundance matrix using the *rarecurve* function in the vegan package (v. 2.5.7) to assess sequencing depth sufficiency. Curves demonstrated that sequencing depth was adequate for most samples, with expected lower-depth curves observed in germ-free and low-biomass samples. Rarefaction was applied to normalize sequencing depth for alpha and beta diversity analyses, and the rarefaction curves are provided in Supplementary Figure S1.

Alpha diversity was assessed using the Chao1 richness index and Shannon diversity index, the latter being less sensitive to variation in sequencing depth [74]. Differences in alpha diversity among groups were evaluated using the Kruskal–Wallis test, followed by Dunn’s post hoc test with false discovery rate (FDR) correction for pairwise comparisons. Beta diversity was assessed using Bray–Curtis dissimilarity matrices and visualized via principal coordinate analysis (PCoA). Confidence ellipses representing 80% of the sample distribution were used to summarize within-group variability without overstating separation among groups. Statistical significance of differences in community composition was tested using permutational multivariate analysis of variance (PERMANOVA) with the adonis2 function (999 permutations) [75]. Differences in alpha and beta diversity indices were considered statistically significant at *p* ≤ 0.05. The indicspecies package (v1.7.9) was used to identify indicator taxa significantly associated with each treatment group [76]. Differential abundance analysis was performed using the DESeq2 package (v1.30.1), with ASVs considered significantly different when the adjusted p-value was less than 0.1 [77]. DESeq2 analyses were performed using unrarefied count data, with DESeq2’s internal median-of-ratios size factor normalization applied to account for differences in sequencing depth. The design formula included diet group as the main factor, and ASVs were considered significantly different at an adjusted *p*-value < 0.1. An adjusted *p*-value cutoff of 0.1 was applied for DESeq2, consistent with exploratory microbiome studies where moderate sample sizes and biological variability justify a slightly less stringent threshold to avoid excluding biologically meaningful taxa.

### 2.6. Statistical Analysis

All morphometric data are expressed as mean ± standard error of the mean (SEM). Statistical differences in body weight, organ parameters, and the relative abundance of bacterial taxa between the control and cheese-supplemented groups were evaluated using paired *t*-tests. A *p*-value of less than 0.05 (*p* < 0.05) was considered statistically significant.

## 3. Results and Discussion

### 3.1. Establishment of Human–Microbiota Associated (HMA) Mice

As previously reported by others, the administration of human fecal material did not negatively affect animal health or performance [12]. In the present study, the body weight of germ-free mice increased progressively from 13.66 ± 0.26 g on day 0 to 15.34 ± 0.08 g on day 7 and 16.86 ± 0.12 g on day 14, indicating normal growth and physiological stability following transplantation. Germ-free baseline samples (BH) exhibited variable and generally low read counts (99–15,921), consistent with low microbial biomass. Human fecal microbiota transplantation successfully resulted in the acquisition of a human-derived gut microbial community in the germ-free mice. After the 14-day stabilization period, fecal samples from HMA mice yielded 15,289–75,069 reads, consistent with microbial transplantation. Although differences were observed in microbial community structure between the HMA mice and the human donor sample (HF), significant similarities were evident in the overall microbial composition (Figures S2–S6). This suggests that members of the human microbiota were able to survive, establish, and stably colonize the murine gut environment. Additionally, we observed that the donor microbiome persisted in the mice’s gut even 6 weeks after fecal transplantation (Figure S6). Although there were some differences at the genus level, at the phylum level, *Bacteroidetes* and *Firmicutes* maintained the same relative abundance in the microbiome after 6 weeks as in the HF inoculum. Our findings are similar to previous reports showing that human fecal transplantation recapitulates the microbial profile observed in the donor sample [19]. Given the reproducibility and sustainability of the transplanted microbiome, the HMA-mice remain a valuable model for the study of microbiome-host interactions.

The use of the HMA mouse model offers several advantages over human-based microbiota studies, including reduced inter-individual variability, control over dietary and environmental factors, smaller sample size requirements, and elimination of confounding host characteristics such as diet, sex, disease state, and immune variability [78,79,80]. Beyond its relevance to translational and therapeutic research, the HMA model serves as a valuable platform for studying gut microbiota dynamics, host–microbe interactions, and diet-induced microbial modulation [12,19,81,82]. In relation to dairy products, both conventional mouse models and human subjects have been used to characterize the impact of milk and fermented dairy foods such as yogurt and kefir on the gut microbiota [12,80,83,84]. However, despite cheese being well recognized for its nutritional and functional properties, its specific influence on gut microbial composition remains less well defined [80,85,86]. To our knowledge, this is the first study to evaluate the effect of Cheddar cheese supplementation on gut microbiota composition using the HMA mouse model.

### 3.2. Microbial Composition of Low-Fat Cheddar Cheese

Since the experimental cheese was manufactured using the starter cultures *Lactococcus lactis*, subsp. *lactis*, and *Streptococcus thermophilus*, viable bacterial populations were quantified using culture-based enumeration. Specifically, *Streptococcus* spp. were enumerated on M17 agar, while total lactic acid bacteria (LAB) were enumerated on de Man, Rogosa, and Sharpe (MRS) agar, both incubated at 37 °C [87]. Enumeration revealed that the cheese contained approximately 8 log CFU/g of *Streptococcus* and total LAB, confirming high microbial viability in the prepared product at the outset and during subsequent refrigerated storage.

**Figure 2:**
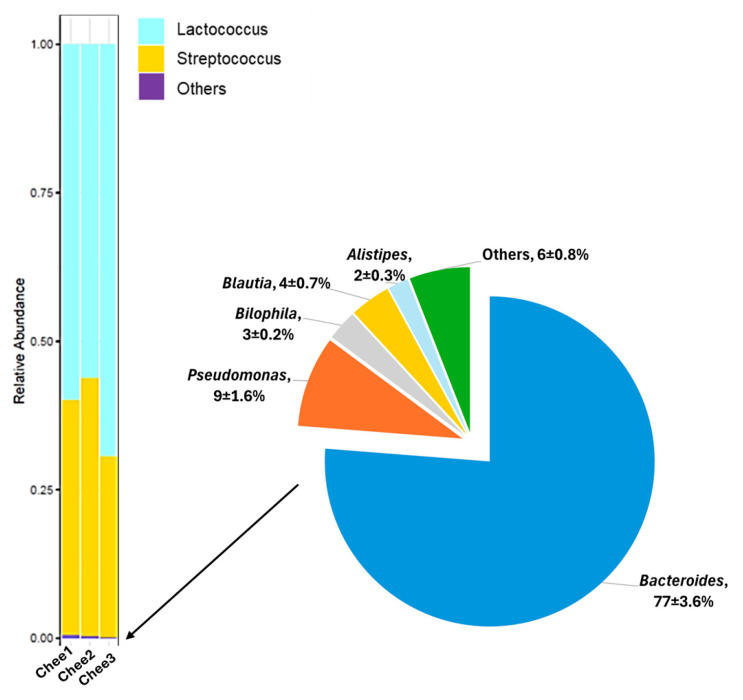
Bacterial community composition of low-fat Cheddar cheese at the genus level. Relative abundance of bacterial genera identified by 16S rRNA gene sequencing in low-fat Cheddar cheese samples. Three independent cheese samples were sequenced, and each column in the relative abundance plot represents an individual replicate.

To further characterize the cheese-associated microbial community, microbial DNA was extracted and subjected to 16S rRNA gene sequencing from three samples (Read depth: 44,165–79,396 reads). The relative abundance of bacterial genera identified in the low-fat Cheddar cheese is presented in Figure 2. The microbiota profile indicated that approximately 99% of the bacterial community was composed of the genera *Lactococcus* (61.8%) and *Streptococcus* (37.8%), consistent with the use of these organisms as starter cultures. The remaining 1% of the microbial population included low-abundance genera, primarily *Bacteroides* and *Pseudomonas*.

### 3.3. Effect of Cheese Supplementation on Body Weight and Organ Indices in HMA Mice

HMA mice were monitored daily for overall health and weighed weekly to assess the effects of dietary treatment on growth. As expected, a steady increase in body weight was observed in both the control and cheese-supplemented groups over the six-week feeding trial. In the cheese group, the mean body weight increased from 16.96 ± 0.26 g at baseline to 24.68 ± 0.63 g at the end of the experiment. A comparable increase was observed in the control group, with no significant difference in final body weights between the groups (Table 2). These findings align with those of Abargouei et al. [88], who reported that increased dairy intake, including milk, low-fat milk, yogurt, and cheese, did not significantly affect body weight, fat mass, or waist circumference in the absence of caloric restriction. Similarly, Sharafetdinov et al. [55] observed that consumption of *Lactobacillus*-fortified probiotic cheese for three weeks reduced body mass index in healthy human subjects compared with controls, while Meyer et al. [89] found an inverse relationship between cheese consumption and obesity prevalence. Consistent with the body weight results, no significant differences were observed in organ weights between the control and cheese-fed groups (Table 3; *p* > 0.05). The lack of difference may be attributed to the isocaloric design of the experimental diets. Furthermore, no gross pathological abnormalities were observed in any animals, indicating that daily low-fat Cheddar cheese supplementation was well tolerated and did not adversely affect general health or organ morphology.

### 3.4. Effect of Low-Fat Cheese on Gut Microbiota in HMA Mice

#### 3.4.1. Sample and Sequencing Outputs

In addition to body weight monitoring, fecal samples were collected from all animals on day 0 (prior to feeding) and at weeks 1 and 6 of the feeding trial (Wk1_control, Wk1_cheese, Wk6_control, and Wk6_cheese). A total of 63 fecal samples were successfully sequenced, and all passed quality control filtering for bacterial community analysis (Table S1). Among mouse fecal samples, sequencing depth in Day 0 control samples ranged from 16,024–99,324 reads, and Day 0 cheese samples ranged from 21,648–60,109 reads. Week 1 control and cheese samples ranged from 15,233–46,663 and 25,664–78,960 reads, respectively. Week 6 control samples ranged from 11,940–68,052 reads, and Week 6 cheese samples ranged from 16,783–45,133 reads. These ranges reflect expected variation in microbial biomass across sample types and confirm adequate sequencing depth for downstream analysis. As described above, low reads were obtained from the germ-free mice samples and thus were removed from the analysis. Following preprocessing, 1836 amplicon sequence variants (ASVs) were identified, representing 2,493,274 high-quality bacterial 16S rRNA gene sequences retained for downstream analyses. Rarefaction curves generated from the ASV abundance table (Figure S1) further demonstrated that sequencing depth was sufficient to capture within-sample bacterial diversity, with lower curves observed only in germ-free and other low-biomass samples.

#### 3.4.2. Low-Fat Cheese Supplementation Significantly Increased Gut Microbial Diversity in HMA Mice

**Figure 3:**
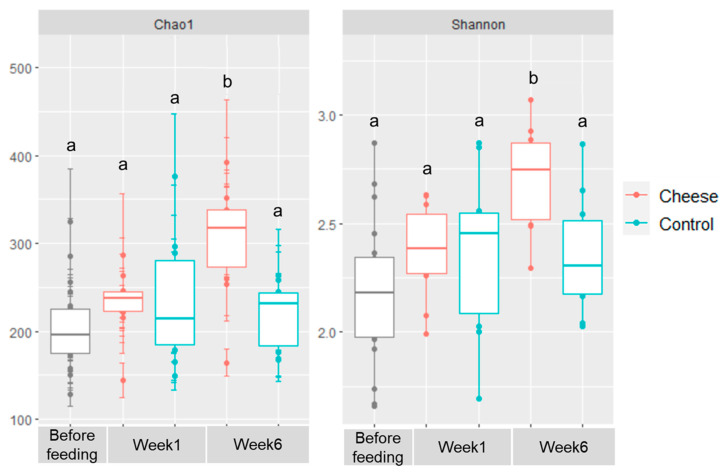
Low-fat Cheddar cheese supplementation increased gut microbial diversity in human microbiota–associated mice. Boxplots show the distribution of gut microbial diversity in control and cheese-fed mice (*n* = 10 animals per group). Diversity was quantified using Chao1 and Shannon indices. The center line represents the median; the box shows the interquartile range (IQR), and whiskers extend to the most extreme values within 1.5 × IQR. A significant increase in diversity was observed in the cheese-fed group (*p* = 0.008) as indicated by the different superscripts in the figure.

Analysis of the alpha diversity of the gut microbiota using the Chao1 richness and Shannon diversity indices revealed that microbial diversity increased progressively in the cheese-fed group throughout the six-week feeding period, whereas it remained relatively unchanged in the control group (Figure 3). Although no significant difference was detected after one week of dietary intervention, sustained cheese supplementation for six weeks led to a significant increase in microbial diversity in the HMA mice (*p* < 0.01). In contrast, the alpha diversity of the control animals remained constant throughout the study period. These results indicate that daily dietary inclusion of low-fat Cheddar cheese, at a level equivalent to two human servings, was associated with increased gut microbial richness and diversity in HMA mice.

**Figure 4:**
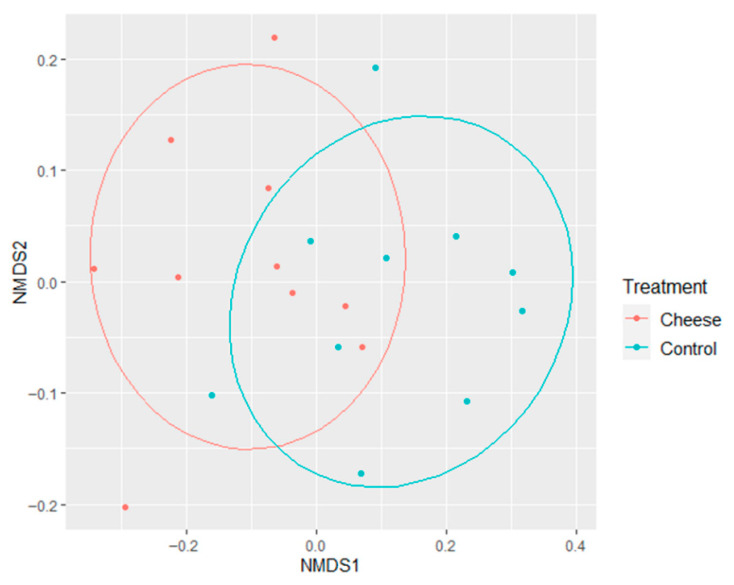
Cheese supplementation reshaped gut microbial community structure in human microbiota–associated mice. Non-metric multidimensional scaling (NMDS) plot based on Bray–Curtis dissimilarity showing compositional differences in fecal microbiota between week 6 control and low-fat Cheddar cheese–fed mice (*R*^2^ = 0.2289, *p* = 0.003). Ellipses represent 80% confidence intervals for each group.

Our findings are consistent with previous studies. Kim et al. [90] reported that consumption of *Lactococcus*-containing cream cheese increased gut microbial diversity in atopic dermatitis mice, while Aljutaily et al. [84] demonstrated that probiotic milk supplementation enhanced microbial diversity in murine models. Further evidence of cheese-induced modulation of the gut microbiota was obtained through non-metric multidimensional scaling (NMDS) analysis based on Bray–Curtis dissimilarity matrices (Figure 4). Distinct clustering patterns indicated that the cheese-fed mice developed microbial communities compositionally different from those of the control animals (*R*^2^ = 0.2289, *p* = 0.003). Because both groups were maintained under identical environmental and husbandry conditions, these differences can be attributed to dietary treatment. Given that the basal diet was double-irradiated and contained negligible microbial content, the changes observed in the gut microbiota of cheese-fed mice are consistent with an effect of dietary cheese inclusion.

Overall, daily supplementation with low-fat Cheddar cheese significantly influenced gut microbial diversity and community structure in HMA mice. Similar findings have been reported for other dairy products such as milk and yogurt [11,18,80]. Ntemiri et al. [91] demonstrated that whole milk supplementation in HMA mice altered gut microbial diversity, while Roy et al. [83] observed no significant change in beta diversity among healthy human volunteers consuming multiple servings of yogurt, emphasizing that large inter-individual variation in human subjects may obscure diet-induced microbial effects. However, greater diversity alone does not necessarily equate to improved host health, and diversity changes should be interpreted in the context of functional outcomes.

### 3.5. Cheese Supplementation Enriched Firmicutes and Beneficial Bacterial Genera in the Gut Microbiota

#### 3.5.1. Phylum-Level Changes

**Figure 5:**
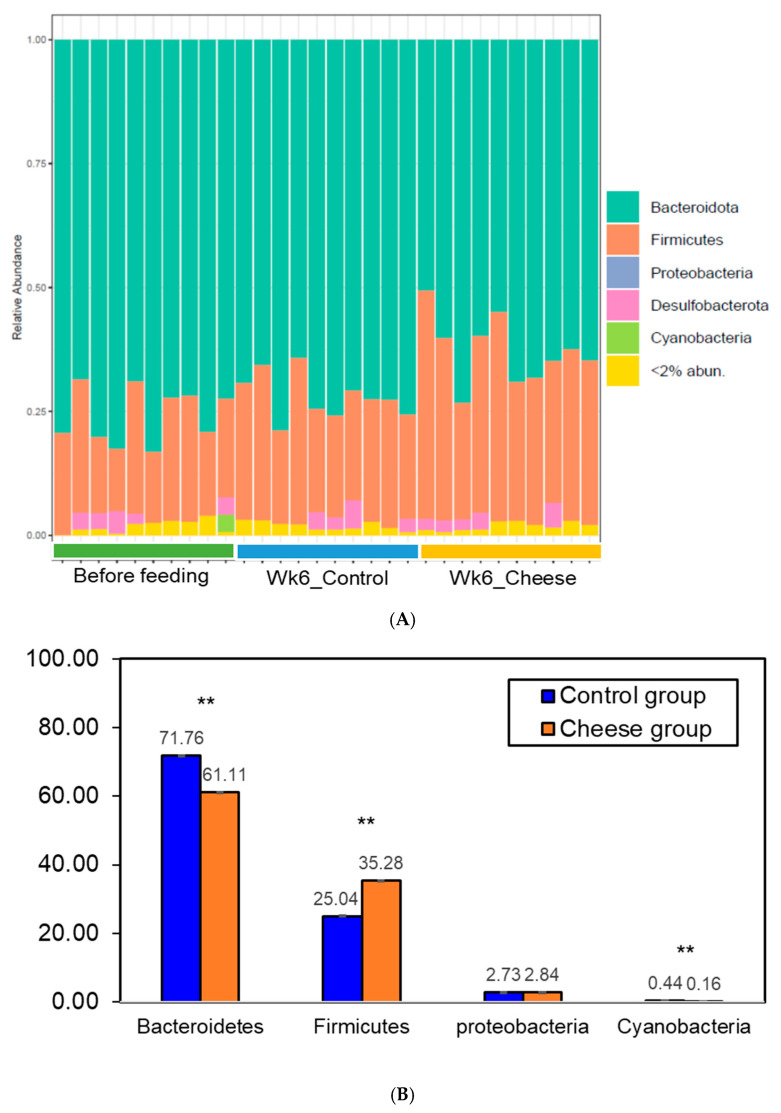
Cheese supplementation altered gut microbial composition at the phylum level in human microbiota–associated mice. (**A**) Relative abundance of bacterial phyla at the start of the feeding trial (Before feeding) and at the end of week 6 in control (Wk6_Control) and cheese-fed (Wk6_Cheese; 7.5% *w*/*w* cheese) mice. (**B**) Predominant bacterial phyla showing significant differences. **: *p* < 0.01.

As previously seen in the HMA mice (Figure S3), *Bacteroidetes* and *Firmicutes* continued to dominate the gut microbial community across the control and cheese groups following the 6-week feeding trial (Figure 5). However, a significant increase in the abundance of *Firmicutes* (25.04% in control vs. 35.28% in cheese group) and a relative decrease in *Bacteroidetes* (71.76% in control vs. 61.11% in the cheese group) were observed (Figure 5B; *p* < 0.05). This increase in the abundance of *Firmicutes* is consistent with an effect of dietary cheese inclusion. Cheddar cheese for this study was prepared using commercial starter cultures consisting of *Lactococcus lactis* subsp. *lactis* and *S. thermophilus*, which belong to the phylum *Firmicutes*. Moreover, as seen from Figure 2, the predominant genera associated with the cheese samples were *Lactococcus* and *Streptococcus*. *Lactococcus* and *Streptococcus* both belong to the family *Streptococcaceae*, and this family was not observed in the gut microbiota of the control animals.

This is significant since members of the phylum *Firmicutes* include taxa that have been widely studied for their potential probiotic functions and associations with host physiology [92]. Many *Firmicutes* are capable of fermenting dietary carbohydrates to produce short-chain fatty acids (SCFAs), metabolites implicated in maintaining intestinal barrier integrity and immune homeostasis [92]. Reduced representation of *Firmicutes* has been reported in association with inflammatory bowel disease (IBD) and stress-related conditions, although these relationships are known to be context dependent [93]. In this study, the observed increase in *Firmicutes* following cheese supplementation is consistent with patterns reported in previous dietary intervention studies, including those examining dairy foods in relation to gut-associated inflammation [52,94]. Together, these findings highlight a meaningful shift in microbial community composition that aligns with established literature, while providing a foundation for future studies to directly assess functional outcomes.

#### 3.5.2. Family-Level Changes

At the family level, *Streptococcaceae*, which includes the two dominant starter cultures used in cheese production (*Lactococcus* and *Streptococcus*), was identified exclusively in the cheese-fed group and not in the control animals. The appearance of *Streptococcaceae* in the gut microbiota of cheese-fed animals but not in controls demonstrates the potential ability of cheese-derived microbes to survive gastric transit and transiently colonize the host gut. Members of this family are known to exert probiotic effects, including pathogen inhibition, enhancement of mucosal immunity, and modulation of the intestinal environment [92]. Other families within *Firmicutes*, such as *Ruminococcaceae*, *Lachnospiraceae*, and *Eubacteriaceae*, were also more abundant in the cheese-fed group, indicating an enhanced potential for SCFA production and complex carbohydrate fermentation, both of which are important for gut health and energy homeostasis [7]. Conversely, *Bacteroidaceae* and *Rikenellaceae*, both under *Bacteroidetes*, showed reduced representation following cheese supplementation.

#### 3.5.3. Genus-Level Changes

**Figure 6:**
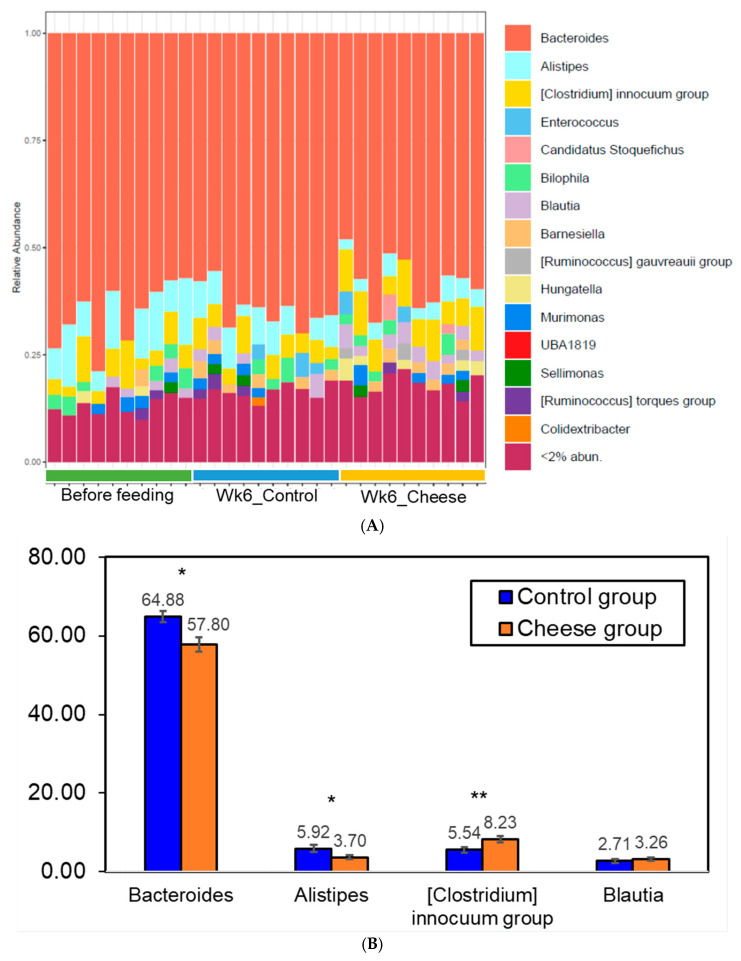
Cheese supplementation altered gut microbial composition at the genus level in human microbiota–associated mice. (**A**) Relative abundance of bacterial genera at the start of the feeding trial (Before feeding) and after six weeks in control (Wk6_Control) and cheese-fed (Wk6_Cheese; 7.5% *w*/*w* cheese) mice. (**B**) Predominant bacterial genera showing changes between groups. *: *p* < 0.05, **: *p* < 0.01.

The change in the relative abundance of genera in the cheese and control groups is depicted in Figure 6. At the genus level, 43 bacterial genera were more abundant in the cheese group when compared to the control group. These include genera associated with the normal human gut microbiota (e.g., *Bilophila*, *Parabacteroides,* and *Anaerostipes*) and some keystone species that are important to balanced immune response, interconnected interactions, and proper homeostasis of the intestinal tract (e.g., *Eubacterium*, *Ruminococcus,* and *Blautia*) [7,95]. On the contrary, 19 genera were less abundant in the cheese group, including bile-tolerant microorganisms such as *Alistipes* and *Bacteroides*, as well as infection-causing agents such as *Enterococcus* [96,97,98].

**Figure 7:**
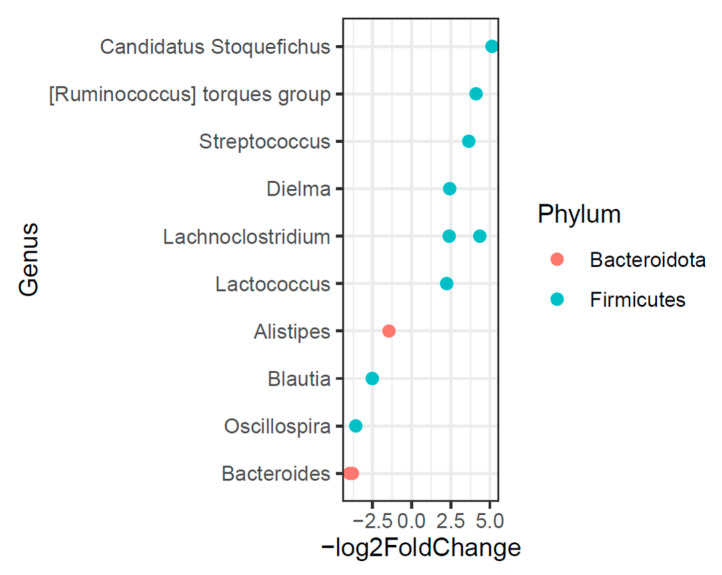
Cheese supplementation altered the differential abundance of bacterial ASVs in human microbiota–associated mice. Differential abundance of bacterial amplicon sequence variants (ASVs) between control and cheese-fed groups was determined using DESeq2 analysis. Each point represents an ASV that significantly differed between groups at an adjusted *p* < 0.1.

Differential abundance analysis using DESeq2 was performed to identify microbial taxa associated with dietary cheese supplementation. In total, 12 ASVs differed significantly between the control and cheese-fed groups (Figure 7). All differentially abundant ASVs corresponded to taxa commonly reported as members of the normal human gut microbiota [99,100,101,102]. Among these, *Oscillospira*, a genus widely detected in both human and animal intestines and often associated with short-chain fatty acid (SCFA) production, was observed at lower relative abundance following cheese supplementation. Similar reductions in *Oscillospira* abundance have been reported in geese fed fermented diets [103]. In contrast, elevated levels of *Oscillospira* have been described in certain animal disease models, including dextran sulfate sodium (DSS)-induced colitis and high-fat diet–induced obesity [104,105], and associations with constipation have also been suggested [106].

Previous studies have reported associations between increased *Alistipes* abundance and various clinical conditions, including cardiovascular disease, depression, and colorectal cancer [102,107,108,109,110], as well as proposed links to inflammatory processes and epithelial barrier alterations [111,112]. However, it is important to note that the biological roles of *Alistipes* and *Oscillospira* remain incompletely defined and appear to be highly context dependent, varying with host physiological state, dietary background, and disease status. Consequently, directional changes in these taxa should be interpreted as compositional shifts rather than indicators of specific pathological or protective outcomes. In contrast, several members of the genus *Lachnoclostridium*, which has been implicated in immune modulation and maintenance of intestinal homeostasis [113], were more abundant in the gut microbiota of cheese-fed mice. Together, these findings highlight diet-associated alterations in gut microbial composition that are consistent with previous dietary intervention studies, while underscoring the need for future work incorporating functional and mechanistic analyses.

#### 3.5.4. ASV-Level and Indicator Species Analysis

For live cultures in the diet to reach the GI tract, they must survive the gastric acid and bile juice. Previous literature has demonstrated that yogurt cultures *Lactobacillus delbrueckii*, subsp. *bulgaricus*, and *S. thermophilus* could be found in the feces of healthy human volunteers after consumption of yogurt [114]. Similar studies also reported that bacterial species in cheese or fermented milk can be transferred to the human gut and colonize in the human gut system [56,59]. Based on the *DESeq2* analysis, both *Streptococcus* and *Lactococcus* were significantly enriched in the gut of the cheese group (Figure 7). In addition, the multipatt function of the *indicspecies* package was used to identify taxa that were significantly associated with each group. The indicator species analysis indicated *Streptococcus* (*p* = 0.001) and *Lactococcus* (*p* = 0.001) were the two major indicator species in the cheese feed group at week 6, which were not found in control animals. The presence of these two genera in the mice microbiota demonstrates the potential ability of cheese microbes to successfully transit the gut and sustain in the intestine [59,115].

#### 3.5.5. Functional and Probiotic Implications

Besides their role in cheese production, *S. thermophilus* and *L. lactis* also exhibit probiotic properties and are associated with promoting host health. Specifically, *S. thermophilus* has been shown to prevent diarrhea and chronic gastritis and exert anti-inflammatory effects by promoting the expression of anti-inflammatory cytokines and exerting antioxidant activity [44,116]. Similarly, *L. lactis* isolated from fermented milk has been reported to exhibit antimicrobial activity against *Salmonella*, *Escherichia*, and *Listeria* [117]. Further, Jaskulski et al. [118] demonstrated the anticarcinogenic potential of *L. lactis* isolated from ricotta cheese. Supplementation of this probiotic was found to promote immune response to the induction of colorectal cancer in rats. Along the same lines, *L. lactis* has also been shown to exert strong anti-proliferative activity against human colon cancer cells in vitro [119]. Thus, in addition to the overall increase in diversity and changes to the microbiota composition, the observed increase in the abundance of cheese cultures in the gut microbiota could play a role in mediating the health benefits associated with cheese consumption.

#### 3.5.6. Study Limitations and Future Directions

While this study provides new insights into how low-fat Cheddar cheese influences the human-derived gut microbiota in an HMA mouse model, several considerations should be noted. The use of a single human donor and only female mice, although common in HMA studies to reduce biological variability, may limit the generalizability of the findings. The study also focused on microbial community composition and did not include functional assessments such as short-chain fatty acid (SCFA) production, inflammatory cytokines, or metabolomic outputs, which would help contextualize the observed microbial shifts. In addition, because cheese was evaluated as a whole food matrix, we did not incorporate additional controls (e.g., heat-treated cheese or starter culture–only preparations) that could help distinguish the relative contributions of viable cheese microbes versus cheese-derived nutrients. These targeted controls, along with direct host functional readouts, represent important next steps. Finally, reliance on 16S rRNA gene sequencing limits both taxonomic and functional resolution; future studies incorporating metagenomic, metabolomic, or predictive functional tools (e.g., PICRUSt2) will enable more direct insight into functional consequences.

## 4. Conclusions

Our results concur with previous literature in demonstrating that the human–microbiota–associated (HMA) mouse model is a robust and translational tool for investigating the effects of diet on the human gut microbiota. In the present study, daily supplementation with low-fat Cheddar cheese, at levels equivalent to two standard servings of low-fat dairy, significantly modulated the gut microbial community structure of HMA mice. Cheese supplementation increased overall microbial diversity, with more than 43 bacterial genera being more abundant in cheese-fed animals than in controls. Notably, *Streptococcus* and *Lactococcus*, the key starter cultures used in cheese production, were markedly enriched in the gut microbiota of cheese-fed mice. The presence of these taxa indicates that viable cheese-associated microbes were able to survive gastrointestinal passage and transiently colonize the host gut.

Given that *Lactococcus* and *Streptococcus* species are known to exhibit probiotic properties, including antimicrobial, anti-inflammatory, and immunomodulatory activities, their increased prevalence may partially explain the reported health-promoting effects of cheese consumption. Overall, these findings provide foundational evidence that low-fat Cheddar cheese can beneficially modulate the gut microbiota and potentially contribute to host well-being through microbial and metabolite-mediated mechanisms. Future research employing integrative multi-omics approaches encompassing transcriptomic, metabolomic, and metagenomic analyses will be critical to fully elucidate the mechanistic pathways underlying the observed diet–microbiota–host interactions and to better define the role of cheese as a complex functional food.

## Figures and Tables

**Table 1:** (**A**) Formulation of the control and cheese-supplemented diets used in the study. (**B**) Proximate composition of the control and cheese-supplemented diets used in the study.

(**A**)		
**Ingredient/g**	**Control Group Diet**	**Cheese Group Diet**
Casein	200	171.4
L-Cystine	3	3
Corn Starch	397.5	400
Maltodextrin	132	132
Sucrose	102.1	95
Cellulose	50	50
Soybean Oil	70	60.6
t-Butylhydroquinone	0.014	0.014
Mineral Mix	3.5	3.5
Calcium Phosphate	-	1.3
Calcium Carbonate	12.5	10
Potassium Citrate	2.5	2.5
Potassium Phosphate	6.9	6.9
Sodium Chloride	2.6	1.3
Vitamin Mix	15	15
Choline Bitartrate	2.5	2.5
Low-fat Cheddar Cheese	-	77.4
Total (g)	1000.1	1032.4
(**B**)		
**Proximate Composition**	**Control Group Diet**	**Cheese Group Diet**
Protein (g%)	18	18
Carbohydrate (g%)	66	66
Fat (g%)	7	7
Protein (kcal)	727	727
Carbohydrate (kcal)	2586	2586
Fat (kcal)	630	630
Total (kcal)	3943	3943
Protein (kcal%)	18	18
Carbohydrate (kcal%)	66	66
Fat (kcal%)	16	16
Calcium (g/kg)	5.1	5.1
Phosphorus (g/kg)	3.1	3.1
Potassium (g/kg)	2.9	3.0
Sodium (g/kg)	1.1	1.1
Magnesium (g/kg)	0.5	0.5
kcal/g	3.9	3.9

**Table 2:** Body weight (g) of control and cheese-fed mice during the six-week feeding trial.

	Week 0	Week 1	Week 2	Week 3	Week 4	Week 5	Week 6
Control	16.97 ± 0.39	19.89 ± 0.55	21.21 ± 0.65	22.23 ± 0.74	24.74 ± 0.94	25.60 ± 0.92	26.01 ± 1.00
Cheese	16.96 ± 0.26	19.65 ± 0.35	21.01 ± 0.31	21.18 ± 0.38	22.98 ± 0.42	23.94 ± 0.56	24.68 ± 0.63

Mean (±SEM) body weight of human microbiota–associated mice fed either a control diet or a diet supplemented with 7.5% (*w*/*w*) low-fat Cheddar cheese. Weights were recorded weekly throughout the six-week feeding period. No significant differences were observed between groups (*p* > 0.05).

**Table 3:** Organ weights (g) of control and cheese-fed mice after the six-week feeding trial.

	Intestine Weight (g)	Colon Weight (g)	Liver Weight (g)	Spleen Weight (g)
Control	1.98 ± 0.09	0.23 ± 0.01	1.27 ± 0.06	0.14 ± 0.02
Cheese	1.91 ± 0.04	0.24 ± 0.01	1.26 ± 0.10	0.08 ± 0.01

Mean (±SEM) organ weights of human microbiota–associated mice fed either a control diet or a diet supplemented with 7.5% (*w*/*w*) low-fat Cheddar cheese. Measurements were recorded at sacrifice following the six-week feeding period. No significant differences were observed between groups (*p* > 0.05).

## Data Availability

The sequencing data generated in this study are available in the NCBI Sequence Read Archive (SRA) under BioProject accession: PRJNA1393523.

## Supplementary Materials

The following supporting information can be downloaded at: https://www.mdpi.com/article/10.3390/foods15010066/s1, Table S1: Sample metadata. Figure S1: Rarefaction curves demonstrating sequencing depth sufficiency across sample groups. Rarefaction curves depicting the relationship between sequencing depth (reads) and the number of observed amplicon sequence variants (ASVs) for all samples. Curves were generated from the unrarefied ASV abundance matrix using the rarecurve function in the vegan package. Samples are colored by category, including mouse diet groups (Day 0, Week 1, and Week 6; control vs. cheese), as well as germ-free mice, human donor samples, and cheese-only samples, to facilitate comparison across sample types. Most curves approach saturation, indicating sufficient sequencing depth, whereas lower curves are observed for low-biomass germ-free samples, as expected. Figure S2: Comparison of bacterial community alpha diversity between human and HMA-mice fecal samples. Boxplots showing alpha diversity of the bacterial communities from human fecal (HF), human microbiota–associated (HMA) mouse (day 14 following fecal transplantation), and AH7 (day 7 following fecal transplant) groups using different diversity indices. The center line represents the median, the box indicates the interquartile range (IQR), and whiskers extend to the most extreme values within 1.5 × IQR of the box. Figure S3: Distinct clustering of bacterial communities among germ-free, humanized, and human fecal samples. Non-metric multidimensional scaling (NMDS) plot based on Bray–Curtis dissimilarity showing beta diversity of fecal microbiota during the humanization process. Samples include germ-free (GF), day 7 after humanization (AH7), human microbiota–associated (HMA; day 14 after humanization), and human fecal inoculum (HF) groups. Distinct clustering with minimal overlap was observed between groups (PERMANOVA, *R*^2^ = 0.6277, *p* = 0.001). Figure S4: Microbial composition of the human donor and germ-free and human microbiota–associated (HMA) mouse fecal samples at the phylum level. (A) Relative abundance of bacterial phyla in the human donor fecal sample and in fecal samples from germ-free and human microbiota–associated (HMA) mice. (B) Major phyla associated with the microbiome of human donor sample and HMA mice fecal samples. ⁎ *p* < 0.05, ⁎⁎ *p* < 0.01, and ⁎⁎⁎ *p* < 0.001. Figure S5: Microbial composition of the human donor and human microbiota–associated (HMA) mouse fecal samples at the genus level. (A) Relative abundance of bacterial genera in the human donor fecal sample and in fecal samples from human microbiota–associated (HMA) mice collected before and after fecal microbiota transplantation. (B) Major genera associated with the microbiome of human donor sample and HMA mice fecal samples. ⁎ *p* < 0.05, ⁎⁎ *p* < 0.01, and ⁎⁎⁎ *p* < 0.001. Figure S6: Microbial composition of human donor fecal sample and mice fecal samples following initial transplantation and at the end of the feeding trial. (A) Relative abundance of human donor sample (HF), and mice fecal samples following transplantation (HMA, 14 days following humanization) and at the end of the feeding trial (Wk6_Control) at the phylum level. (B) Relative abundance of HF, HMA, and Wk6_Control at the genus level.
