# Supplementary material for: Low-Fat Cheddar Cheese Influences Gut Microbiota Composition and Diversity in Human Microbiota–Associated Mice
Source: Foods. 2025 Dec 25;15(1):66. doi: 10.3390/foods15010066 (PMC12785681; doi:10.3390/foods15010066)

Table S1. Sample metadata

Sample ID	Experiment	Group	Time	Sample detail
D14afterhumanize1.AH01Dec2020	Humanization	Humanization	D14	Day14_Humanization
D14afterhumanize10.AH10Dec2020	Humanization	Humanization	D14	Day14_Humanization
D14afterhumanize11.AH11Dec2020	Humanization	Humanization	D14	Day14_Humanization
D14afterhumanize12.AH12Dec2020	Humanization	Humanization	D14	Day14_Humanization
D14afterhumanize13.AH13Dec2020	Humanization	Humanization	D14	Day14_Humanization
D14afterhumanize14.AH14Dec2020	Humanization	Humanization	D14	Day14_Humanization
D14afterhumanize15.AH15Dec2020	Humanization	Humanization	D14	Day14_Humanization
D14afterhumanize16.AH16Dec2020	Humanization	Humanization	D14	Day14_Humanization
D14afterhumanize17.AH17Dec2020	Humanization	Humanization	D14	Day14_Humanization
D14afterhumanize18.AH18Dec2020	Humanization	Humanization	D14	Day14_Humanization
D14afterhumanize19.AH19Dec2020	Humanization	Humanization	D14	Day14_Humanization
D14afterhumanize2.AH02Dec2020	Humanization	Humanization	D14	Day14_Humanization
D14afterhumanize20.AH20Dec2020	Humanization	Humanization	D14	Day14_Humanization
D14afterhumanize3.AH03Dec2020	Humanization	Humanization	D14	Day14_Humanization
D14afterhumanize4.AH04Dec2020	Humanization	Humanization	D14	Day14_Humanization
D14afterhumanize5.AH05Dec2020	Humanization	Humanization	D14	Day14_Humanization
D14afterhumanize6.AH06Dec2020	Humanization	Humanization	D14	Day14_Humanization
D14afterhumanize7.AH07Dec2020	Humanization	Humanization	D14	Day14_Humanization
D14afterhumanize8.AH08Dec2020	Humanization	Humanization	D14	Day14_Humanization
D14afterhumanize9.AH09Dec2020	Humanization	Humanization	D14	Day14_Humanization
Day7humanize1.7H01Dec2020	Humanization	Humanization	D7	Day7_Humanization

Day7humanize10.7H10Dec2020	Humanization	Humanization	D7	Day7_Humanization
Day7humanize11.7H11Dec2020	Humanization	Humanization	D7	Day7_Humanization
Day7humanize12.7H12Dec2020	Humanization	Humanization	D7	Day7_Humanization
Day7humanize13.7H13Dec2020	Humanization	Humanization	D7	Day7_Humanization
Day7humanize14.7H14Dec2020	Humanization	Humanization	D7	Day7_Humanization
Day7humanize15.7H15Dec2020	Humanization	Humanization	D7	Day7_Humanization
Day7humanize16.7H16Dec2020	Humanization	Humanization	D7	Day7_Humanization
Day7humanize17.7H17Dec2020	Humanization	Humanization	D7	Day7_Humanization
Day7humanize18.7H18Dec2020	Humanization	Humanization	D7	Day7_Humanization
Day7humanize19.7H19Dec2020	Humanization	Humanization	D7	Day7_Humanization
Day7humanize2.7H02Dec2020	Humanization	Humanization	D7	Day7_Humanization
Day7humanize20.7H20Dec2020	Humanization	Humanization	D7	Day7_Humanization
Day7humanize3.7H03Dec2020	Humanization	Humanization	D7	Day7_Humanization
Day7humanize4.7H04Dec2020	Humanization	Humanization	D7	Day7_Humanization
Day7humanize5.7H05Dec2020	Humanization	Humanization	D7	Day7_Humanization
Day7humanize6.7H06Dec2020	Humanization	Humanization	D7	Day7_Humanization
Day7humanize7.7H07Dec2020	Humanization	Humanization	D7	Day7_Humanization
Day7humanize8.7H08Dec2020	Humanization	Humanization	D7	Day7_Humanization
Day7humanize9.7H09Dec2020	Humanization	Humanization	D7	Day7_Humanization
be4humanize1.BH01Dec2020	Humanization	Humanization	D0	Day0_Humanization
be4humanize10.BH10Dec2020	Humanization	Humanization	D0	Day0_Humanization
be4humanize11.BH11Dec2020	Humanization	Humanization	D0	Day0_Humanization
be4humanize12.BH12Dec2020	Humanization	Humanization	D0	Day0_Humanization

be4humanize2.BH02Dec2020	Humanization	Humanization	D0	Day0_Humanization
be4humanize20.BH20Dec2020	Humanization	Humanization	D0	Day0_Humanization
be4humanize3.BH03Dec2020	Humanization	Humanization	D0	Day0_Humanization
be4humanize8.BH08Dec2020	Humanization	Humanization	D0	Day0_Humanization
be4humanize13.BH13Dec2020	Humanization	Humanization	D0	Day0_Humanization
be4humanize14.BH14Dec2020	Humanization	Humanization	D0	Day0_Humanization
be4humanize16.BH16Dec2020	Humanization	Humanization	D0	Day0_Humanization
be4humanize18.BH18Dec2020	Humanization	Humanization	D0	Day0_Humanization
be4humanize19.BH19Dec2020	Humanization	Humanization	D0	Day0_Humanization
be4humanize4.BH04Dec2020	Humanization	Humanization	D0	Day0_Humanization
be4humanize5.BH05Dec2020	Humanization	Humanization	D0	Day0_Humanization
be4humanize7.BH07Dec2020	Humanization	Humanization	D0	Day0_Humanization
be4humanize9.BH09Dec2020	Humanization	Humanization	D0	Day0_Humanization
humanfeces1.HF01Dec2020	Humanization	Humanization	Human feces	Human feces
humanfeces2.HF02Dec2020	Humanization	Humanization	Human feces	Human feces
D0cheese1.D0T01Dec2020	Cheeseexp	Cheese	Day0	Beforefeeding
D0cheese10.D0T10Dec2020	Cheeseexp	Cheese	Day0	Beforefeeding
D0cheese2.D0T02Dec2020	Cheeseexp	Cheese	Day0	Beforefeeding
D0cheese3.D0T03Dec2020	Cheeseexp	Cheese	Day0	Beforefeeding
D0cheese4.D0T04Dec2020	Cheeseexp	Cheese	Day0	Beforefeeding
D0cheese5.D0T05Dec2020	Cheeseexp	Cheese	Day0	Beforefeeding
D0cheese6.D0T06Dec2020	Cheeseexp	Cheese	Day0	Beforefeeding
D0cheese7.D0T07Dec2020	Cheeseexp	Cheese	Day0	Beforefeeding

D0cheese8.D0T08Dec2020	Cheeseexp	Cheese	Day0	Beforefeeding
D0cheese9.D0T09Dec2020	Cheeseexp	Cheese	Day0	Beforefeeding
D0control1.D0C01Dec2020	Cheeseexp	Control	Day0	Beforefeeding
D0control10.D0C10Dec2020	Cheeseexp	Control	Day0	Beforefeeding
D0control2.D0C02Dec2020	Cheeseexp	Control	Day0	Beforefeeding
D0control3.D0C03Dec2020	Cheeseexp	Control	Day0	Beforefeeding
D0control4.D0C04Dec2020	Cheeseexp	Control	Day0	Beforefeeding
D0control5.D0C05Dec2020	Cheeseexp	Control	Day0	Beforefeeding
D0control6.D0C06Dec2020	Cheeseexp	Control	Day0	Beforefeeding
D0control7.D0C07Dec2020	Cheeseexp	Control	Day0	Beforefeeding
D0control8.D0C08Dec2020	Cheeseexp	Control	Day0	Beforefeeding
D0control9.D0C09Dec2020	Cheeseexp	Control	Day0	Beforefeeding
Wk1cheese1.W1T01Dec2020	Cheeseexp	Cheese	Wk1	Wk1_Cheese
Wk1cheese10.W1T10Dec2020	Cheeseexp	Cheese	Wk1	Wk1_Cheese
Wk1cheese2.W1T02Dec2020	Cheeseexp	Cheese	Wk1	Wk1_Cheese
Wk1cheese3.W1T03Dec2020	Cheeseexp	Cheese	Wk1	Wk1_Cheese
Wk1cheese4.W1T04Dec2020	Cheeseexp	Cheese	Wk1	Wk1_Cheese
Wk1cheese5.W1T05Dec2020	Cheeseexp	Cheese	Wk1	Wk1_Cheese
Wk1cheese6.W1T06Dec2020	Cheeseexp	Cheese	Wk1	Wk1_Cheese
Wk1cheese7.W1T07Dec2020	Cheeseexp	Cheese	Wk1	Wk1_Cheese
Wk1cheese8.W1T08Dec2020	Cheeseexp	Cheese	Wk1	Wk1_Cheese
Wk1cheese9.W1T09Dec2020	Cheeseexp	Cheese	Wk1	Wk1_Cheese
Wk1control1.W1C01Dec2020	Cheeseexp	Control	Wk1	Wk1_Control

Wk1control10.W1C10Dec2020	Cheeseexp	Control	Wk1	Wk1_Control
Wk1control2.W1C02Dec2020	Cheeseexp	Control	Wk1	Wk1_Control
Wk1control3.W1C03Dec2020	Cheeseexp	Control	Wk1	Wk1_Control
Wk1control4.W1C04Dec2020	Cheeseexp	Control	Wk1	Wk1_Control
Wk1control5.W1C05Dec2020	Cheeseexp	Control	Wk1	Wk1_Control
Wk1control6.W1C06Dec2020	Cheeseexp	Control	Wk1	Wk1_Control
Wk1control7.W1C07Dec2020	Cheeseexp	Control	Wk1	Wk1_Control
Wk1control8.W1C08Dec2020	Cheeseexp	Control	Wk1	Wk1_Control
Wk1control9.W1C09Dec2020	Cheeseexp	Control	Wk1	Wk1_Control
Wk6cheese1.W6T01Dec2020	Cheeseexp	Cheese	Wk6	Wk6_Cheese
Wk6cheese10.W6T10Dec2020	Cheeseexp	Cheese	Wk6	Wk6_Cheese
Wk6cheese2.W6T02Dec2020	Cheeseexp	Cheese	Wk6	Wk6_Cheese
Wk6cheese3.W6T03Dec2020	Cheeseexp	Cheese	Wk6	Wk6_Cheese
Wk6cheese4.W6T04Dec2020	Cheeseexp	Cheese	Wk6	Wk6_Cheese
Wk6cheese5.W6T05Dec2020	Cheeseexp	Cheese	Wk6	Wk6_Cheese
Wk6cheese6.W6T06Dec2020	Cheeseexp	Cheese	Wk6	Wk6_Cheese
Wk6cheese7.W6T07Dec2020	Cheeseexp	Cheese	Wk6	Wk6_Cheese
Wk6cheese8.W6T08Dec2020	Cheeseexp	Cheese	Wk6	Wk6_Cheese
Wk6cheese9.W6T09Dec2020	Cheeseexp	Cheese	Wk6	Wk6_Cheese
Wk6control1.W6C01Dec2020	Cheeseexp	Control	Wk6	Wk6_Control
Wk6control10.W6C10Dec2020	Cheeseexp	Control	Wk6	Wk6_Control
Wk6control2.W6C02Dec2020	Cheeseexp	Control	Wk6	Wk6_Control
Wk6control3.W6C03Dec2020	Cheeseexp	Control	Wk6	Wk6_Control

Wk6control4.W6C04Dec2020	Cheeseexp	Control	Wk6	Wk6_Control
Wk6control5.W6C05Dec2020	Cheeseexp	Control	Wk6	Wk6_Control
Wk6control6.W6C06Dec2020	Cheeseexp	Control	Wk6	Wk6_Control
Wk6control7.W6C07Dec2020	Cheeseexp	Control	Wk6	Wk6_Control
Wk6control8.W6C08Dec2020	Cheeseexp	Control	Wk6	Wk6_Control
Wk6control9.W6C09Dec2020	Cheeseexp	Control	Wk6	Wk6_Control
cheesesupplement.CHEE1Dec2020	Cheeseexp	Others	Cheese	Chee_sup
cheesesupplement2.CHEE2Dec2020	Cheeseexp	Others	Cheese	Chee_sup
cheesesupplement3.CHEE3Dec2020	Cheeseexp	Others	Cheese	Chee_sup

Figure S1. Rarefaction curves demonstrating sequencing depth sufficiency across sample groups. Rarefaction curves depicting the relationship between sequencing depth (reads) and the number of observed amplicon sequence variants (ASVs) for all samples. Curves were generated from the unrarefied ASV abundance matrix using the rarecurve function in the vegan package. Samples are colored by category, including mouse diet groups (Day 0, Week 1, and Week 6; control vs. cheese), as well as germ-free mice, human donor samples, and cheese-only samples, to facilitate comparison across sample types. Most curves approach saturation, indicating sufficient sequencing depth, whereas lower curves are observed for low-biomass germ-free samples, as expected.

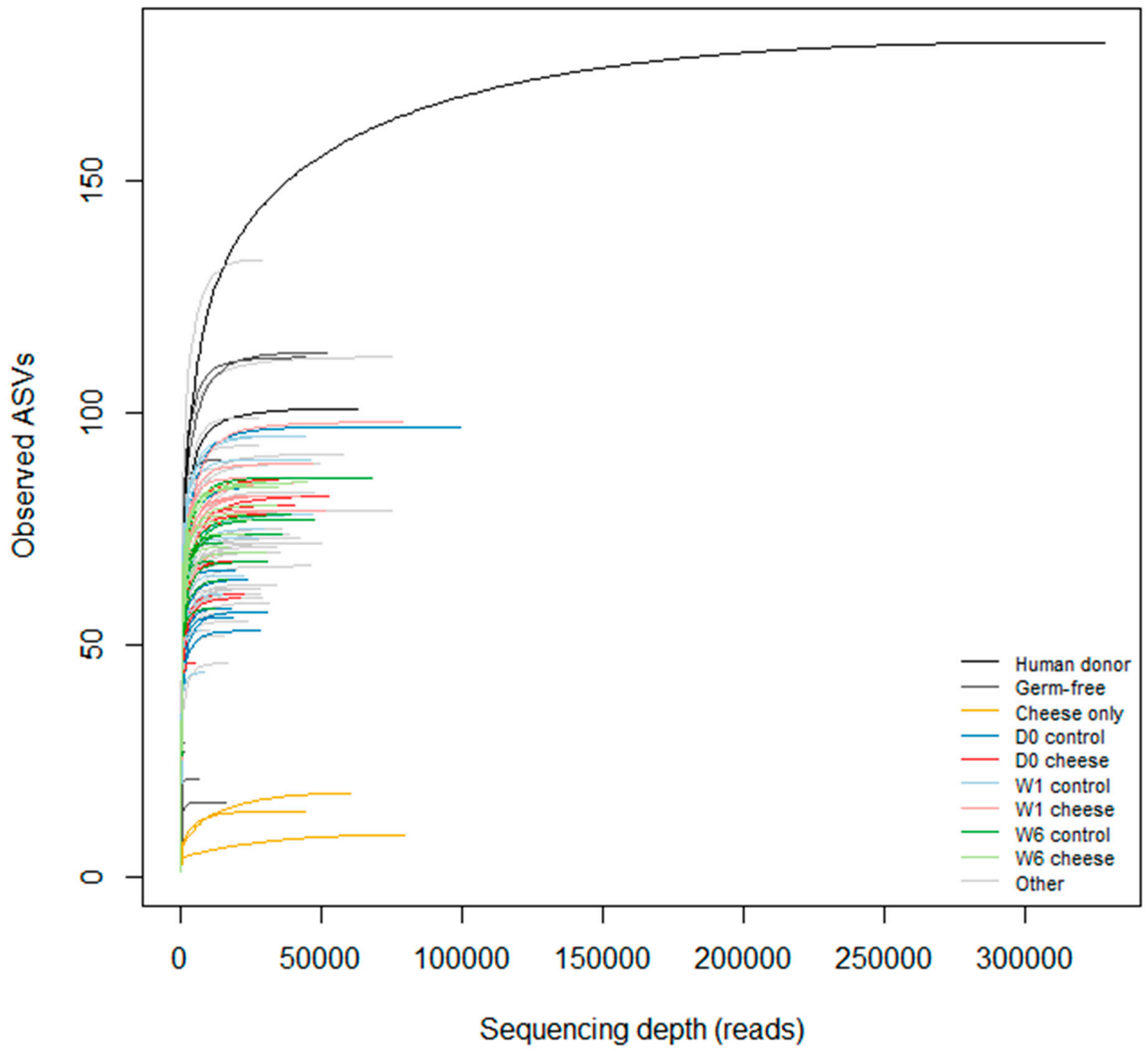

Figure S2. Comparison of bacterial community alpha diversity between human and HMA-mice fecal samples. Boxplots showing alpha diversity of the bacterial communities from human fecal (HF), human microbiota–associated (HMA) mouse (day 14 following fecal transplantation), and AH7 (day 7 following fecal transplant) groups using different diversity indices. The center line represents the median, the box indicates the interquartile range (IQR), and whiskers extend to the most extreme values within 1.5 × IQR of the box.

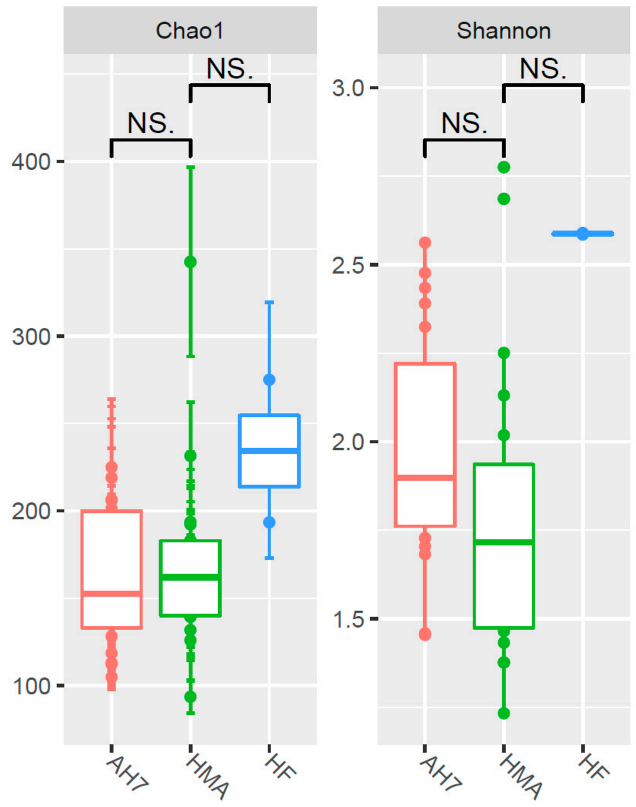

Figure S3. Distinct clustering of bacterial communities among germ-free, humanized, and human fecal samples. Non-metric multidimensional scaling (NMDS) plot based on Bray–Curtis dissimilarity showing beta diversity of fecal microbiota during the humanization process. Samples include germ-free (GF), day 7 after humanization (AH7), human microbiota–associated (HMA; day 14 after humanization), and human fecal inoculum (HF) groups. Distinct clustering with minimal overlap was observed between groups (PERMANOVA, $R^2 = 0.6277$, $p = 0.001$).

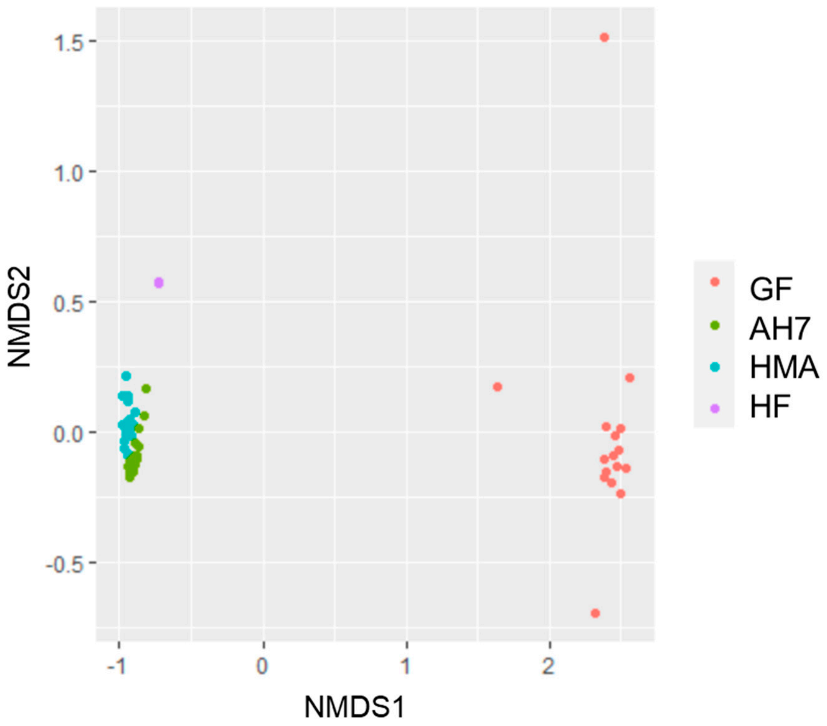

Figure S4. Microbial composition of the human donor and germ-free and human microbiota–associated (HMA) mouse fecal samples at the phylum level. (A) Relative abundance of bacterial phyla in the human donor fecal sample and in fecal samples from germ-free and human microbiota–associated (HMA) mice. (B) Major phyla associated with the microbiome of human donor sample and HMA mice fecal samples. ∗ $p < 0.05$, ∗∗ $p < 0.01$, and ∗∗∗ $p < .001$.

(A)

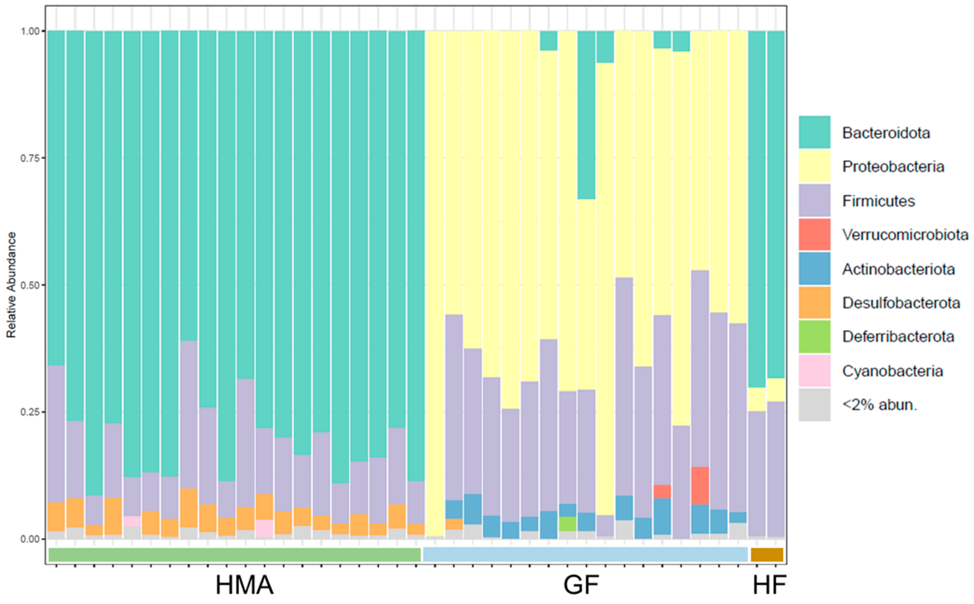

(B)

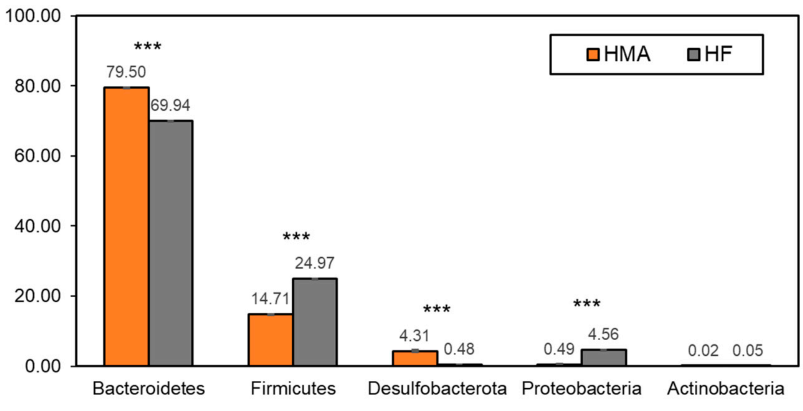

Figure S5. Microbial composition of the human donor and human microbiota–associated (HMA) mouse fecal samples at the genus level. (A) Relative abundance of bacterial genera in the human donor fecal sample and in fecal samples from human microbiota–associated (HMA) mice collected before and after fecal microbiota transplantation. (B) Major genera associated with the microbiome of human donor sample and HMA mice fecal samples. $* \ p < 0.05$, $** \ p < 0.01$, and $*** \ p < 0.001$.

(A)

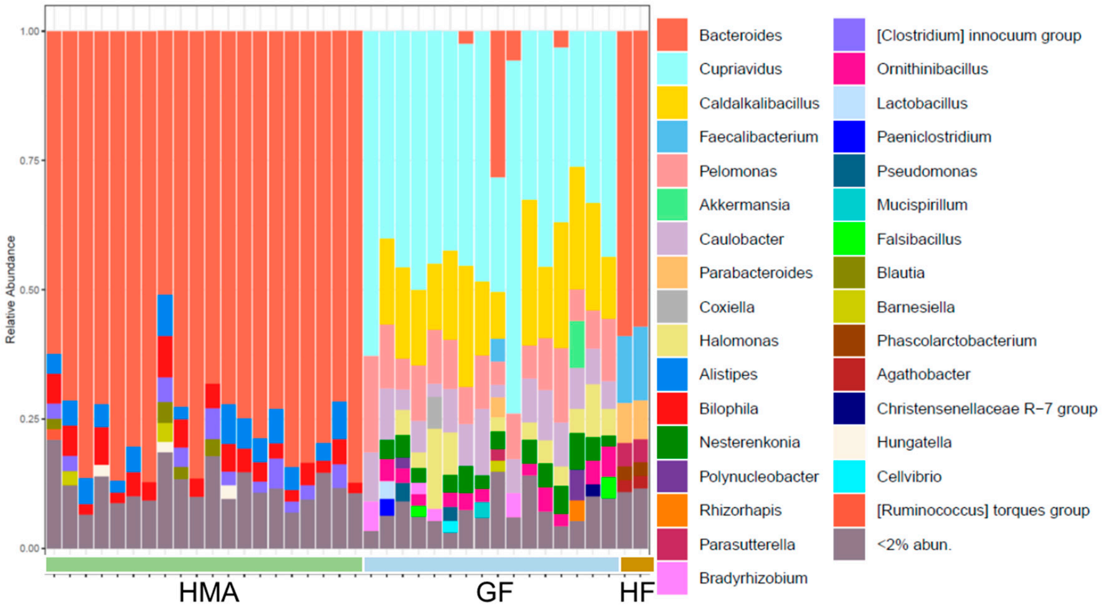

(B)

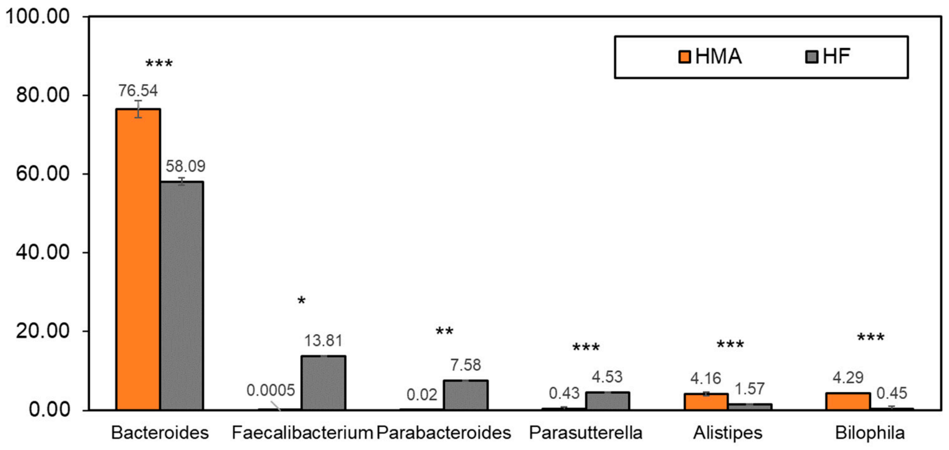

Figure S6. Microbial composition of human donor fecal sample and mice fecal samples following initial transplantation and at the end of the feeding trial. (A) Relative abundance of human donor sample (HF), and mice fecal samples following transplantation (HMA, 14 days following humanization) and at the end of the feeding trial (Wk6_Control) at phylum level. (B) Relative abundance of HF, HMA and Wk6_Control at genus level.

(A)

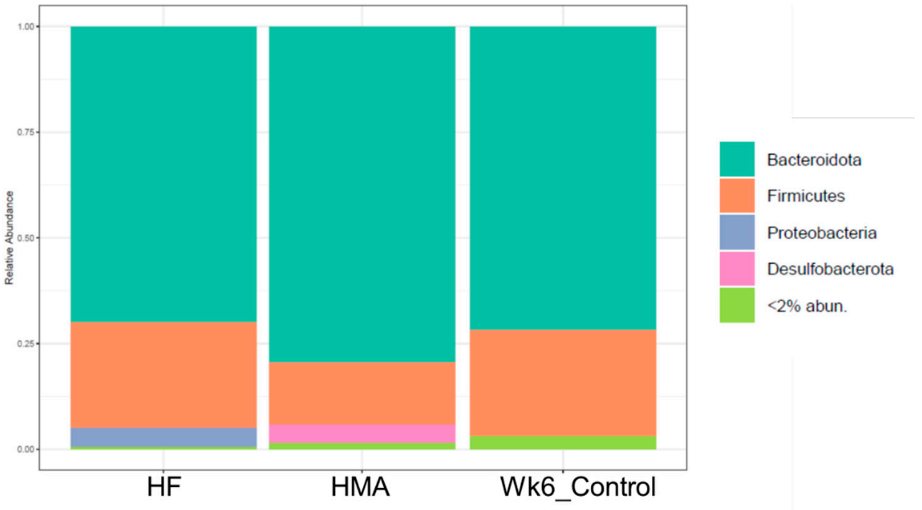

(B)

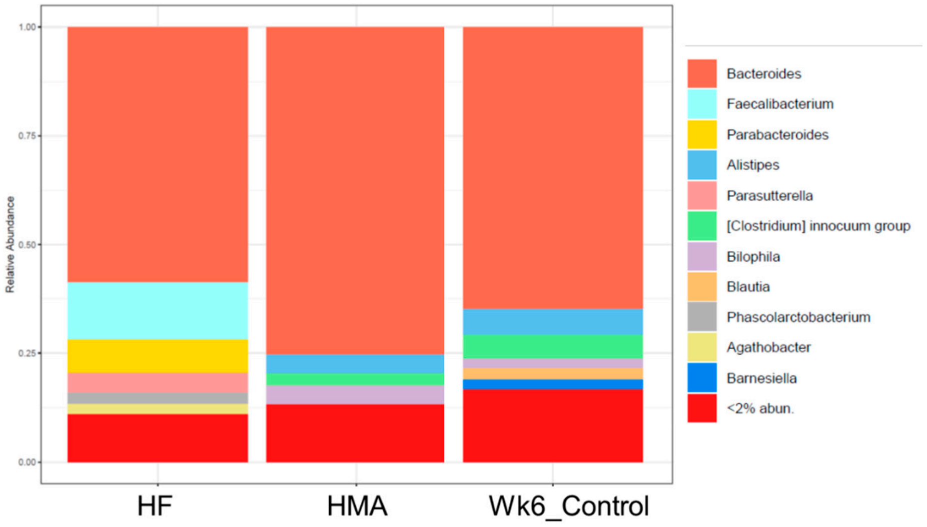

Supplement: Supplementary file 1 [file foods-15-00066-s001.zip › foods-4015336-supplementary.pdf]
